# Oxidative stress-induced NCC activation in the development of nocturnal polyuria in mice: Therapeutic potential of a sustained hydrogen-releasing silicon-based agent

**DOI:** 10.1016/j.bbrep.2025.101923

**Published:** 2025-01-20

**Authors:** Yosuke Sekii, Hiroshi Kiuchi, Kentaro Takezawa, Norichika Ueda, Takahiro Imanaka, Sohei Kuribayashi, Koichi Okada, Shinichiro Fukuhara, Ryoichi Imamura, Hiromistu Negoro, Yuki Kobayashi, Hikaru Kobayashi, Norio Nonomura

**Affiliations:** a Department of Urology, Graduate School of Medicine, Osaka University, Suita, Japan; b Department of Urology, Nagasaki University Graduate School of Biomedical Sciences, Nagasaki, Japan; c Department of Urology, Institute of Medicine, University of Tsukuba, Tsukuba, Japan; d The Institute of Scientific and Industrial Research, Osaka University, Suita, Japan

**Keywords:** NCC, Nocturia, Nocturnal polyuria, Oxidative stress, Silicon, Sodium, Urinary sodium excretion

## Abstract

Nocturnal polyuria is a prevalent condition associated with significant deterioration in quality of life and increased risk of mortality. Despite its clinical relevance, the underlying pathogenesis is poorly understood, and existing therapies have limited efficacy. A recent study in mouse model revealed that overactivation of the intrarenal SPAK (STE20/SPS1-related proline–alanine rich protein kinase)–sodium chloride co-transporter (NCC) pathway in the distal renal tubule is a crucial mechanism contributing to nocturnal polyuria. Here, we demonstrate that increased oxidative stress in the kidney activates the NCC, leading to insufficient sodium excretion during the active period and compensatory sodium excretion during the inactive period, resulting in polyuria during the inactive period. In addition, we show that a newly developed antioxidant—a silicon component agent—reduced oxidative stress and inhibited NCC activation, resulting in the amelioration of polyuria during the inactive period. These findings highlight the critical contributions of intrarenal oxidative stress to the pathogenesis of nocturnal polyuria and suggest that silicon-based agent holds promise for clinical application as a novel treatment for nocturnal polyuria.

## Highlights

• Increased renal oxidative stress activates the NCC.
• Activation of the NCC impairs urinary sodium excretion.
• Compensatory sodium excretion results in diurnal polyuria.
• A silicone-based agents improved diurnal polyuria.

## Graphical abstract

Increased oxidative stress in the kidney activates NCC and suppresses salt excretion, resulting in nocturnal polyuria. Our novel Silicon-based agent consistently produces a high amount of hydrogen in the intestine, which reduces renal oxidative stress, thus improving nocturnal polyuria.

**Figure:**
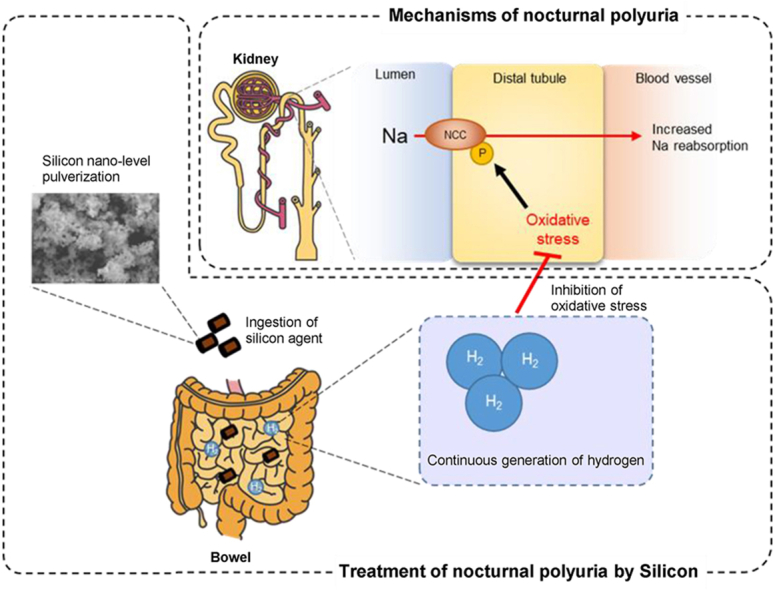

## 1 Introduction

Nocturia is a common condition characterized by the need to wake up to pass urine during the main sleep period [1]; it affects 65 % of people over the age of 50 [2], with two or more nocturia episodes compromising quality of life [3]. Recently, large-scale epidemiological studies have demonstrated that nocturia is associated with a 1.5-fold increase for one urination versus zero, 1.9-fold for two urinations, and 2.1-fold for three or more urinations [4]. The etiology of nocturia include bladder storage disorders [5], sleep disorder r [6], nocturnal polyuria [7], and combinations of these disorders. Nocturnal polyuria accounts for 50–80 % of nocturia cases [8]; therefore, management of nocturnal polyuria is indispensable for the treatment of nocturia. However, currently available drug therapies have limited efficacy for nocturnal polyuria, and the precise molecular mechanisms remain unknown. To dissect the underlying pathophysiology and identify therapeutic targets for this disease, we recently established an animal model that reflects human conditions [9]. Given that advancing age [10] and dietary salt intake [11] are well-established risk factors for nocturnal polyuria, our model combines a high-salt diet with the pharmacological inhibition of nitric oxide synthase using Nω-nitro-l-arginine methyl ester (l-NAME), to replicate the age-related decline in nitric oxide production. Our study revealed that overactivation of the intrarenal angiotensin II-SPAK-NCC pathway (SPAK, STE20/SPS1-related proline–alanine rich protein kinase; NCC, chloride co-transporter), which regulates the sodium homeostasis located in the distal tubule of the kidney, plays a crucial role in nocturnal polyuria. Overactivation of this pathway results in impaired excretion of sodium during the active phase and compensatory sodium excretion during the inactive phase. Excessive sodium excretion during the inactive phase leads to nocturnal polyuria due to osmotic diuresis, indicating that the angiotensin II-SPAK-NCC pathway may be a promising therapeutic target. In addition to angiotensin II, other known activators of this pathway include aldosterone, insulin, potassium, and oxidative stress [12,13]. Among them, oxidative stress is responsible for a wide variety of age-associated diseases, including cancer, infection, and arteriosclerosis-associated diseases. Moreover, evidence suggests that biological stress exerts profound effects on human health, influencing both physiological and psychological processes [14]. Therefore, we hypothesize that increased oxidative stress in the kidneys associated with aging may confer NCC activation, resulting in nocturnal polyuria.

**Table 1:** Primer sequences for the quantitative real-time PCR amplification.

Gene	Forward (5′–3′)	Reverse (5′–3′)
p22phox	TGCCAGTGTGATCTATCTGCT	TCGGCTTCTTTCGGACCTCT
gp91phox	GCGGTGTGCAGTGCTATCAT	GCGGTGTGCAGTGCTATCAT
p47phox	CCTGCCACTTAACCAGGAACA	CCTGCCACTTAACCAGGAACA
Gapdh	TGTGTCCGTCGTGGATCTGA	TTGCTGTTGAAGTCGCAGGAG

Hydrogen—well known for its antioxidant properties [15]—is promising for the treatment of oxidative stress-associated diseases and has been used for myocardial infarction [16] and diabetes [17] in clinical research. However, effective and continuous transport of hydrogen into the body requires large-scale equipment because of the highly volatile nature of hydrogen. We recently developed a novel hydrogen-producing silicon-based agent (Si-based agent) that is orally ingested and features sustained hydrogen generation in the intestine [18].The efficacy of Si-based agents has been reported in animal models of Parkinson’s disease [19], renal injury [20], and male infertility [21] through reduction in oxidative stress. In this study, we aimed to demonstrate the role of oxidative stress in nocturnal polyuria and evaluate the efficacy of a Si-based agent novel antioxidant on nocturnal polyuria in a mouse model.

## 2 Materials and methods

### 2.1 Ethics declarations

All animal experiments were performed in accordance with the Osaka University Guidelines for the Care and Use of Laboratory Animals and were approved by the Osaka University Animal Care and Use Committee (No. J006580-013). This study complied with all the ethical regulations. This study was performed in accordance with ARRIVE guidelines.

### 2.2 Animals

Nineteen-week-old C57BL/6J mice were used in this study (SLC Japan, Tokyo, Japan). The mice were housed under a 12:12-h light-dark cycle with controlled humidity and temperature and free access to food pellets and tap water.

Mice were randomly assigned into three groups: the control group, which was fed a normal salt diet (NSD; 0.2 % wt/wt NaCl); NP model group, which was given a high-salt diet (1 % HSD; 1 % wt/wt NaCl) + l-NAME in drinking water for 2 weeks, as previously described [9] (5 mg/dL, N5751, Sigma-Aldrich, St. Louis, MO, USA); and the NP model treated with a Si-based agent group, which received 1 % HSD diet containing a 1 % wt silicon-based agent + l-NAME in drinking water for 2 weeks. All meals were purchased from Oriental Yeast (Tokyo, Japan). A Si-based agent was fabricated from Si powder (Koujundo Chemical Laboratory Si 3 N Powder, ∼5 μm). The Si powder was milled using the bead milling method.

### 2.3 Measurement of urine volume and time

Urine volume and timing were assessed using the automated voided stain-on-paper (aVSOP) method, as previously described [22]. In brief, a roll of laminated filter paper, pretreated to change color to dark purple upon contact with urine, was advanced beneath a water-repellent wire grid at a constant rate of 10 cm/h. Mice were individually housed in cages measuring 110 mm × 160 mm × 75 mm (H × D × W) for a period of four days. Urination events were recorded and quantified using ImageJ software (version 1.53e, National Institutes of Health, USA), enabling conversion to urine volume.

Given the nocturnal nature of mice, the diurnal polyuria index (DPi), defined as the ratio of diurnal urine volume to total daily urine volume, was calculated. This index served as a corresponding measure for nocturnal polyuria observed in humans.

### 2.4 Urine analysis of mice

Mice were individually housed in metabolic cages (Techniplast, Tokyo, Japan) with ad libitum access to food and water. Urine was subsequently collected for analysis. Due to the limited urine output during the inactive (daytime) period, the daytime urine volume and sodium concentration were calculated as previously described [9]. Specifically, the inactive period urine volume was determined using the formula: (24-h urine volume) − (active period urine volume). Similarly, the inactive period urinary sodium concentration was calculated as: [(24-h urinary sodium content) − (active period urinary sodium content)]/(inactive period urine volume). Urine samples were sent to Fujifilm PET Systems Corporation (Tokyo, Japan) to analyze urinary sodium concentrations using the electrode method. Calibration of the analyzer was conducted as required to ensure measurement accuracy.

### 2.5 Histological evaluation of kidney tissue

Mice were euthanized during the active phase via isoflurane inhalation. The kidneys were harvested, fixed in 4 % paraformaldehyde, and subsequently embedded in paraffin. Histological analyses were performed using hematoxylin-eosin (H&E) staining. Proximal and distal tubules were differentiated based on distinct morphological characteristics. Proximal tubules were identified by their open lumen surrounded by cuboidal or low columnar epithelial cells with abundant eosinophilic cytoplasm and prominent brush borders. In contrast, distal tubules appeared smaller, more closely packed, with cells exhibiting less cytoplasmic volume and an absence of brush borders.

### 2.6 Quantitative real-time PCR

cDNA was synthesized using the Prime Script RT Reagent Kit (Perfect Real Time) (Takara Bio, Shiga, Japan) according to the manufacturer’s protocol. Quantitative real-time PCR was conducted on a QuantStudio 7 Flex System (Applied Biosystems, Foster City, CA, USA) with PowerUp SYBR Green Master Mix (Applied Biosystems, Foster City, CA, USA). Relative gene expression levels were normalized to the housekeeping gene *Gapdh* and calculated using the delta-delta Ct method. The primer sequences used for quantitative real-time PCR are listed in Table 1.

### 2.7 Immunohistochemistry staining and ROS scoring

Immunohistochemistry was performed on 4-μm-thick sections of formalin-fixed, paraffin-embedded kidney tissue. Sections were deparaffinized after heating at 68 °C for 20 min. The primary antibody used was rabbit anti-4-hydroxynonenal (4-HNE, 1:300, Bioss). Antibodies were diluted in DAKO REAL Antibody Diluent (Agilent, Santa Clara, CA, USA) and incubated with the sections overnight at 4 °C. Detection was carried out using the DAKO EnVision Kit (Agilent, Santa Clara, CA, USA) following the manufacturer’s protocol.

Reactive oxygen species (ROS) expression levels were semi-quantified using immunostaining for 4-hydroxynonenal (4-HNE). For each animal, three randomly selected fields were analyzed. The number of 4-HNE-positive cells and the total number of glomeruli in each field were counted. The percentage of 4-HNE-positive cells relative to each glomerulus was calculated, and the final ROS score for each sample was determined as the average percentage across the three fields.

### 2.8 Immunoblotting

Whole kidney proteins were extracted by homogenization in lysis buffer and centrifuged, and the whole lysate and membrane fractions were prepared in sodium dodecyl sulfate (SDS) sample buffer (Cosmo Bio, Tokyo, Japan). Protein concentrations were determined using the Lowry method, and samples were stored at −80 °C until analysis. A total of 20 μg of protein per sample was separated using either 8 % non-SDS-PAGE or 10 % SDS-PAGE, followed by transfer to membranes for immunoblotting. Membranes were blocked with Blocking One (Nacalai Tesque, Kyoto, Japan) and incubated with primary antibodies overnight at 4 °C in Tris-buffered saline. Detection was achieved using an HRP-linked anti-rabbit IgG antibody (Cell Signaling Technology, Danvers, MA, USA) and Chemi-Lumi One reagent (Nacalai Tesque, Kyoto, Japan).

Blots were imaged using the ChemiDoc XRS Plus imaging system (Bio-Rad, Hercules, CA, USA), and band intensities were quantified using ImageJ software. Primary antibodies included rabbit anti-NCC (1:1000, Millipore) and rabbit anti-phosphorylated NCC (Thr53, 1:1000, Phospho Solution). The secondary antibody was HRP-conjugated anti-rabbit IgG (1:5000, Cell Signaling Technology).

### 2.9 Statistical analysis

Results are given as mean ± SEM, and *P*-values <0.05 were considered statistically significant. Two-tailed Student's *t-test* for experiments with two groups and one-way ANOVA with Tukey-Kramer post-hoc analysis was used to compare results across the three groups. F-statistics and partial eta-squared (ηp^2^) values were reported to indicate effect sizes. All analyses were performed using the JMP software (SAS Institute, Cary, NC, USA) or GraphPad Prism 8.0 (GraphPad Software, SD, CA, USA).

## 3 Results

### 3.1 Renal oxidative stress increased in nocturnal polyuria model

**Fig. 1:**
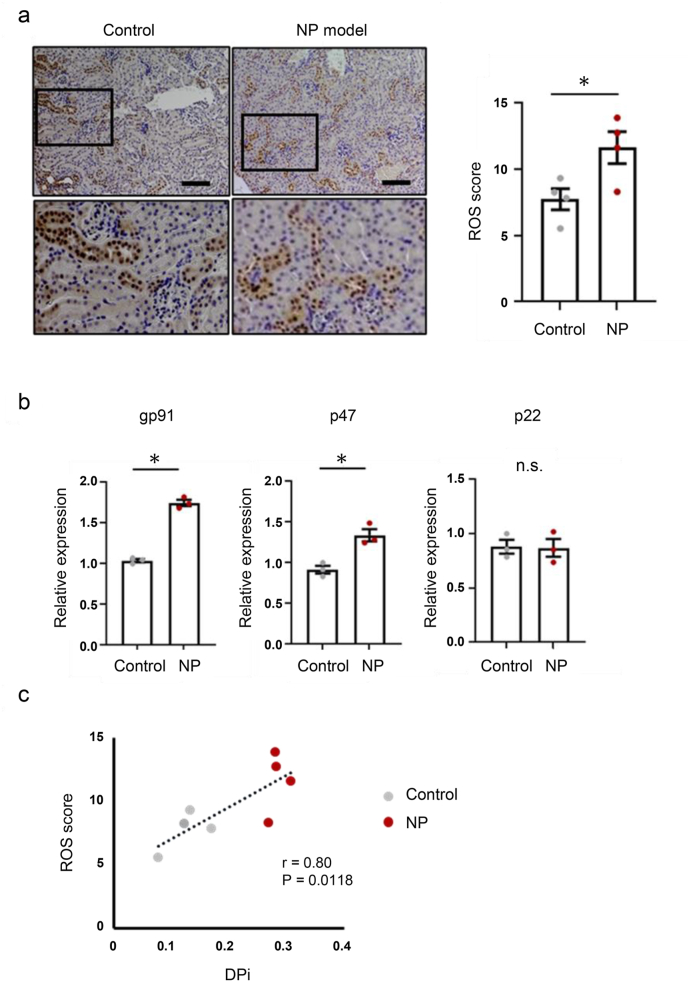
Renal oxidative stress in nocturnal polyuria mice was increased. **a.** Representative immunostaining of renal 4-HNE and ROS score in the control group and nocturnal polyuria model group. Original magnification: 200×. Scale bar, 100 μm. **b.** Relative mRNA expression levels of kidney tissue *gp91phox*, *p22phox*, and *p47phox* genes as assessed by quantitative real-time PCR in control and NP model groups. **c.** Association between ROS score and diurnal polyuria index (DPi). DPi = diurnal urine volume/daily urine volume.

To evaluate the association between renal oxidative stress and nocturnal polyuria, the expression of reactive oxygen species (ROS) in the kidney was compared between control mice and a mouse model of nocturnal polyuria (NP model). Immunohistochemical analysis showed that the ROS marker, 4-HNE positive cells were abundant in the distal convoluted tubule, and 4-HNE expression was significantly higher in the NP model than in control mice (Fig. 1a). To evaluate the transcriptional levels of ROS markers, we examined the mRNA expression of gp41, p47, and p22 subunits of NADPH oxidase, which controls the pathways regulating ROS production. Of these, the expression of gp41 and p47 was significantly higher in NP model than in control (Fig. 1b). Next, we evaluated the relationship between ROS and DPi, the ratio of inactive to daily urine output corresponding to human nocturnal polyuria, and found that DPi was correlated with the ROS score (Fig. 1c), suggesting that diurnal polyuria is associated with renal oxidative stress.

### 3.2 A Si-based agent decreased oxidative stress, and improved diurnal polyuria in mice

**Fig. 2:**
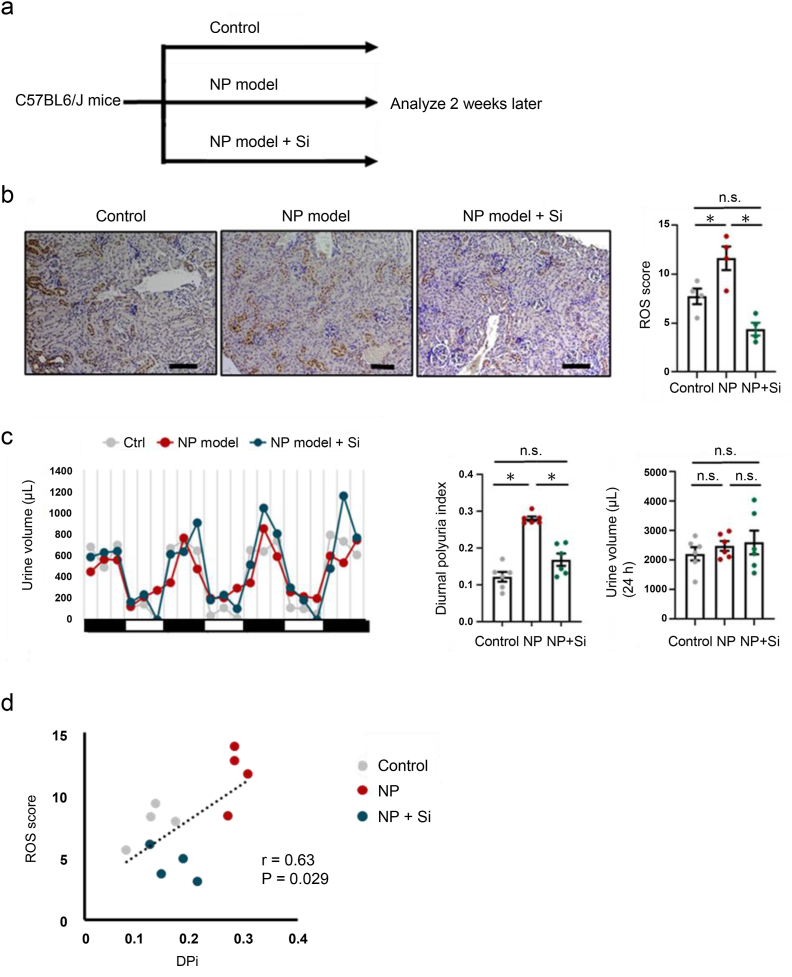
A Si-based agent improved diurnal polyuria index in mice **a.** Experimental protocol. **b.** Representative immunostaining of renal 4-HNE and ROS score in the control group, nocturnal polyuria model group (NP model), and nocturnal polyuria model + Si group (NP model + Si). Original magnification: 200×. Scale bar, 100 μm. Statistical analysis was performed using one-way ANOVA with Tukey-Kramer post-hoc analysis. F (2, 9) = 15.6, *P* = 0.0012, ηp^2^ = 0.78. ∗*P* < 0.05. **c.** Mean 4-h urinary volume (left) for consecutive 3 days measured by aVSOP in control (grey) and NP model mice (red) and NP model + Si (blue). DPi (diurnal polyuria index = diurnal urine volume/daily urine volume) (middle) and daily urine volume (right). Statistical analysis was performed using one-way ANOVA with Tukey-Kramer post-hoc analysis. F (2, 15) = 39.65, *P* < 0.001, ηp^2^ = 0.84 for DPi, and F (2, 15) = 0.49, *P* = 0.62, ηp^2^ = 0.06 for daily urine volume. ∗*P* < 0.05. **d.** Association between ROS score and DPi.

First, we tested whether a Si-based agent reduced ROS levels in the kidney. The mice were divided into three groups: control, NP model, and NP with Si supplementation (Fig. 2a). Immunohistochemistry of 4-HNE in the kidney revealed that the increased ROS score in the NP model decreased after the administration of Si agents (Fig. 2b). Next, we evaluated the influence of ROS reduction in the kidneys on urine production. After the administration of silicone components in the NP model, 24 h urine volume did not change (*P* = 0.793); however, the urine output in the inactive periods significantly decreased, resulting in a decrease in DPi (0.28 vs 0.17, *P* < 0.01) (Fig. 2c). A correlation between ROS score and DPi was found among the control, NP model, and NP + Si agents (Fig. 2d). These results suggest that the Si-based agent improved diurnal polyuria in the NP model.

### 3.3 Mechanisms underlying the amelioration of diurnal polyuria in mice: reduced phosphorylation of NCC by a Si-based agent

**Fig. 3:**
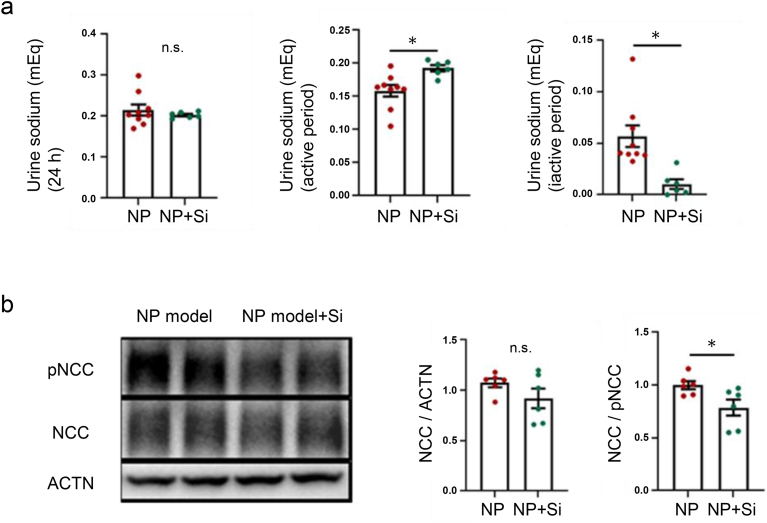
Molecular mechanisms of oxidative stress on nocturnal polyuria and therapeutic effects of a Silicon-based agent. **a.** Urinary sodium excretion per day (left) and active (middle) and inactive (right) periods. ∗*P* < 0.05. **b.** Representative immunoblotting (left) and quantitative analysis (right) of renal phosphorylated NCC, NCC after Si-based agent administration. ∗*P* < 0.05.

To address the mechanisms underlying the reduction in urine production during the inactive period in the NP model after Si administration, we first examined urinary sodium excretion during the active and inactive phase. Si administration in the NP model did not alter 24 h urinary sodium excretion but increased urinary sodium excretion in the active period; additionally, it decreased sodium excretion in the inactive period (Fig. 3a). Next, to investigate the molecular mechanisms underlying the changes in urinary sodium excretion, we evaluated the expression of NCC, an essential regulator of sodium balance in the distal tubule. Total NCC expression was not different between the NP model and NP model + Si, whereas the expression of phosphorylated NCC—the active form of NCC—decreased after Si administration (0.99 vs 0.77, *P* < 0.05) (Fig. 3b). Taken together, these findings suggest that elevated renal oxidative stress enhances sodium retention via activation of NCC, contributing to nocturnal polyuria. The administration of the Si agent was shown to attenuate oxidative stress, inhibit NCC activation, and promote sodium excretion during the active periods, thereby mitigating nocturnal polyuria.

## 4 Discussion

Together, these results provide novel evidence that increased oxidative stress in the kidney activates NCC, leading to insufficient sodium excretion during the active period and compensatory sodium excretion during the inactive period, resulting in polyuria during the inactive period. A novel silicone-based agents improved diurnal polyuria by reducing the intrarenal oxidative stress.

Nocturnal polyuria is prevalent in middle-aged and elderly people and is the most common cause of nocturia, which is associated with the impaired quality of life and increased mortality [4,11], and its underlying etiology remains insufficiently understood. Recent studies have highlighted the critical role of sodium in the pathogenesis of this condition. Excessive salt intake has been identified as a significant risk factor for the development of nocturnal polyuria, and salt restriction ameliorates its severity [23,24]. Furthermore, elevated sodium consumption is associated with the development of lower leg edema, which in turn contributes to nocturnal polyuria [25]. In a recent study, we investigated urinary sodium excretion in patients undergoing nephrectomy [26], and found that nocturnal polyuria was associated with a postoperative increase in nocturnal sodium excretion. These results indicated that a shift in salt excretion from daytime to nighttime is a crucial mechanism underlying nocturnal polyuria. In the present study, elevated renal oxidative stress activated NCC, which was accompanied by reduced urinary sodium excretion during the active periods and increased urinary sodium excretion during the inactive periods, reflecting a shift in sodium excretion. Conversely, when oxidative stress was alleviated by the administration of the silicon-based agent, the sodium excretion pattern normalized, with increased sodium excretion during the active periods. These observations suggested that the shift in sodium excretion plays a crucial role in the pathogenesis of nocturnal polyuria.

Sodium homeostasis is achieved by strict regulation of excretion and reabsorption in the kidneys. Ninety-nine percent of the sodium filtered through the glomerulus is reabsorbed in the tubules: 65–75 % in proximal tubules, 25 % in Henle's loop, 5 % in distal tubules, and 1–2% in the collecting duct [27]. Sodium channels in the distal tubule and collecting duct reabsorb only a small amount of sodium; however, they are considered the most significant in determining sodium reabsorption [28]. NCC in the distal tubules and epithelial sodium channel (ENaC) in the collecting ducts are channels that mainly regulate sodium reabsorption in each tubule, and NCC contributes to the pathogenesis of nocturnal polyuria. The inactive form of NCC is phosphorylated to its active form to reabsorb sodium in the distal tubule. SPAK is the protein involved in the phosphorylation of NCC [29], and its activation is modulated by factors such as angiotensin II, aldosterone, hyperinsulinemia, extracellular potassium levels and oxidative stress [12,13]. In this study, we focused on oxidative stress due to its well-documented contribution to a variety of age-associated diseases. A growing number of studies has demonstrated that oxidative stress directly mediates the phosphorylation of SPAK [13,30,31]. In mouse distal tubular cells, oxidative stress triggered by nitric oxide (NO) depletion leads to the phosphorylation of SPAK, which subsequently drives the phosphorylation of NCC [13]. Notably, pharmacological inhibition of SPAK phosphorylation effectively prevents this cascade, highlighting the pivotal role of oxidative stress-induced SPAK phosphorylation. Similarly, studies in ventricular and pulmonary epithelial cells have shown that oxidative stress induces SPAK phosphorylation, followed by downstream phosphorylation events [30,31]. When cells derived from SPAK-knockout mice are utilized, these subsequent phosphorylation processes are suppressed, further substantiating the critical involvement of SPAK in oxidative stress-mediated signaling pathways. To confirm that oxidative stress was involved in nocturnal polyuria, we investigated ROS levels in the NP model and found a significant increase in ROS at both the protein and mRNA levels in the kidney, concomitant with NCC activation. Notably, when renal oxidative stress was mitigated by antioxidant treatment in the NP mouse model, NCC activation was suppressed, resulting in enhanced sodium excretion and a corresponding reduction in polyuria during the inactive phase. These results indicate that ROS in the kidneys alter the sodium excretion rhythm, and cause thereby contributing to the pathophysiology of nocturnal polyuria.

Our previous study demonstrated the activation of the NCC in this nocturnal polyuria model not only during the active phase but also during the inactive phase [9]. We observed a reciprocal relationship between sodium excretion in these phases, suggesting that impaired sodium excretion during the active phase may lead to compensatory excretion during the inactive phase. This compensatory mechanism is plausible given the relatively lower sodium excretion rates during the inactive period compared to the active period. In this study, oxidative stress increased during the active phase, and it seems unlikely that oxidative stress rapidly fluctuates within a single day. The persistently elevated oxidative stress likely contributes to NCC activation during both phases, leading to insufficient sodium excretion in the active phase and compensatory sodium excretion in the inactive phase. This shift in sodium excretion is postulated to result in an increase in diurnal urine volume. Further studies are needed to confirm this hypothesis.

Biomarker studies related to lower urinary tract symptoms have identified several candidates, including nerve growth factor (NGF), adenosine triphosphate (ATP), and C-reactive protein (CRP) [32]. These biomarkers have been explored in the context of overactive bladder, providing insights into potential diagnostic and therapeutic targets. In addition, the utility of exosomal noncoding RNAs in renal diseases has been demonstrated in conditions such as IgA nephropathy [33] and diabetic nephropathy [34]. For instance, in IgA nephropathy, five candidate miRNAs (hsa-miR-146b-3p, hsa-miR-599, hsa-miR-4532, hsa-miR-664b-5p, and hsa-miR-221-5p) were identified as potential biomarkers. These miRNAs distinguished IgA nephropathy cases from controls with an AUC >0.90, and the presence of all five miRNAs was associated with 100 % sensitivity and specificity for diagnosing IgA nephropathy. While this study focused on oxidative stress, advanced computational techniques, including in silico methods such as molecular docking studies and AI-based approaches like machine learning and deep learning, offer significant potential to identify novel etiological agents and therapeutic compounds. These techniques enable efficient analysis of complex datasets and examination of a wide range of candidate substances in a cost-effective and time-efficient manner. Future research leveraging these computational tools could complement experimental studies, providing valuable insights into disease mechanisms and accelerating the discovery of effective interventions for oxidative stress and nocturnal polyuria.

Hydrogen is effective for the treatment of several diseases in clinical research and is a promising agent owing to its anti-oxidative stress properties. The inhalation of hydrogen gas and consumption of saturated hydrogen water are the most common methods of hydrogen ingestion. However, inhalation of hydrogen gas for chronic diseases is difficult to conduct at home because hydrogen gas inhalation machines have safety and portability issues for home use. Therefore, we developed an innovative silicon component agent that generates hydrogen in the body, enabling sufficient and continuous hydrogen generation without the need for equipment. Previously, the administration of silicon components ameliorated kidney damage [20] and improve impaired sperm motility with varicose veins [21] without any side effects. In the present study, treatment with the Si-based agent effectively reduced oxidative stress in the kidneys and suppressed NCC activation, thereby normalizing the shift in urinary sodium excretion. As a result, the NP model with Si-based agent exhibited an increase in active-periods urine volume. These results suggested that Si-based agent is a promising clinical treatment for nocturnal polyuria. Silicon has been widely studied for its contributions to medical research, particularly in areas involving oxidative stress, such as Parkinson's disease, stroke, and diabetes. Its ability to generate hydrogen through ingestion has been explored as a mechanism for mitigating oxidative damage. Additionally, mesoporous silica nanoparticles have shown potential as drug delivery carriers, especially for targeting the brain [35]. Beyond medical applications, silicon is also investigated for its role in protecting plants from pathogens, further highlighting its versatility in research fields [36].

Conventional antioxidants such as vitamin C, vitamin E, and coenzyme Q10 differ in several ways from silicon-based hydrogen, an antioxidant generated by the Si-based agent. Vitamin C, being water-soluble, acts extracellularly and is excreted in the urine, which limits its ability to provide sustained antioxidant effects. Vitamin E and coenzyme Q10, both fat-soluble, are absorbed with lipids and act intracellularly after uptake, providing antioxidant benefits. However, long-term consumption of these compounds carries minor risks. For example, excessive accumulation of vitamin E has been associated with adverse effects such as bleeding, fatigue, and diarrhea [37]. Similarly, long-term ingestion of coenzyme Q10, though rare, has been reported to cause gastric discomfort [38]. In contrast, molecular hydrogen is unique in its dual intracellular and extracellular antioxidant properties, owing to its small molecular size. The Si-based agent continuously generates hydrogen within the gastrointestinal tract, enabling sustained antioxidant activity. Furthermore, hydrogen selectively neutralizes hydroxyl radicals, the most toxic oxidants, without interfering with other reactive oxygen species that have physiological roles [39]. While silicon dioxide and silicone are already widely used as food additives with established safety profiles, the long-term effects of ingesting silicon-based agents require further investigation. Comprehensive studies, particularly in large animal models, are essential to assess potential complications and ensure safety prior to clinical application. Additionally, research focusing on optimal dosage and efficacy in comparison to conventional antioxidants will be critical to support the translational potential of this innovative therapeutic approach.

This study has several limitations. First, the extent of hydrogen production by Si-based agents within the body was not investigated in this study. However, previous *in vitro* study has demonstrated that silicone formulations produce significant amounts of hydrogen [18]. In animal study, Si-based agents have been shown to increase the concentration of hydrogen in the blood [20]. Second, the mechanism by which hydrogen reaches the kidneys after its generation in the intestine has not been thoroughly elucidated. It is hypothesized that hydrogen may reach the kidneys either by dissolving in the bloodstream and being delivered through systemic circulation, or by direct diffusion to renal tissues. Further research is required to delineate these potential pathways. Third, while this silicone formulation has been shown to produce no apparent adverse effects when administered to animals in the dosages used in this study, its effects on the human body are not yet known. Additional studies are required to evaluate its safety and efficacy in humans before clinical application.

## 5 Conclusions

This study provides new insights into the pathogenesis of nocturnal polyuria and potential therapeutic targets. We identified a molecular mechanism in which increased renal oxidative stress impairs sodium excretion via activation of NCC, leading to nocturnal polyuria. Furthermore, our silicon-based agent attenuated renal oxidative stress, and holds promise for clinical application as a novel treatment for nocturnal polyuria.

## Author contributions

Yosuke Sekii and Hiroshi Kiuchi were responsible for experimental work throughout the study. Yosuke Sekii: Methodology, Investigation, Data curation, and writing of the original draft. Hiroshi Kiuchi: Conceptualization, Supervision, Writing – review, and editing. Kentaro Takezawa: Investigation. Norichika Ueda: Investigation. Takehiro Imanaka: Investigation. Sohei Kuribayashi: Investigation. Koichi Okada: Investigation. Shinichiro Fukuhara: Conceptualization, Supervision. Roichi Imamura: Investigation. Hiromistu Negoro: Investigation. Yuki Kobayashi: Developed and provided Si-based agents; Hikaru Kobayashi: Developed and provided Si-based agents. Norio Nonomura: Conceptualization, Supervision.

## Funding information

This work was supported by the Grant-in-Aid for Scientific Research from the Japan Society for the Promotion of Science (JSPS) (Grant number: 22K16789) and the 10.13039/501100009033Center of Innovation Program (COI Program Grant Number JPMJCE1310).

## Declaration of competing interest

The authors declare that they have no known competing financial interests or personal relationships that could have appeared to influence the work reported in this paper.

## Data Availability

The data that support the findings of this study are available from the corresponding author upon reasonable request.
